# A first genetic linage map construction and QTL mapping for growth traits in *Larimichthys polyactis*

**DOI:** 10.1038/s41598-020-68592-0

**Published:** 2020-07-15

**Authors:** Feng Liu, Wei Zhan, Qingping Xie, Honglin Chen, Bao Lou, Wantu Xu

**Affiliations:** 1 Institute of Hydrobiology, Zhejiang Academy of Agricultural Sciences, Shiqiao Road 198, Hangzhou, 310021 China; 2 Xiangshan Gangwan Aquatic Seeds Co., Ltd, Ningbo, 315700 China

**Keywords:** Next-generation sequencing, Genetic linkage study, Quantitative trait

## Abstract

*Larimichthys polyactis* is a commercially important marine fish species in Eastern Asia, yet very few genetic resources exist. In particular, genetic linkage maps are critical tools for genetic breeding. In this study, we generated a high resolution linkage map from a family of 110 individuals and their parents by resequencing the individuals. 3,802 effective SNPs were mapped to 24 linkage groups (LGs). The map spanned 2,567.39 cm, with an average marker interval of 0.66 cm. We used the map to conduct QTL analysis for growth traits, and found 31 markers were significantly associated with growth-related traits. Specifically, three SNPs were identified for total length, nineteen SNPs for body length, and nine SNPs for body weight. The identified SNPs could explain 15.2–22.6% of the phenotypic variation. SNPs associated with growth traits were distributed on LG6 and LG11, and candidate genes included, *kif26b*, *bat1*, *gna1*, *gbgt1*, and *amfr*, which may regulate growth. The linkage map and mapped QTLs would be useful for improving the quality of *L. polyactis* via marker-assisted selection.

## Introduction

The small yellow croaker, *Larimichthys polyactis*, is a benthopelagic marine fish species that is endemic to the Bohai Sea, Yellow Sea and East China Sea^1,2^, and is an ecologically and commercially important marine fish species^3^. Artificial propagation of *L. polyactis* was achieved in 2015^4^, and has contributed to the increased success of farming of this species in China. However, genetic studies of *L. polyactis* are limited to population structure and genetic diversity surveys^5–7^, and transcriptome analysis under specific conditions^8^, and there are few established microsatellite markers^9–11^, and mitochondrial genome sequences that are available^12,13^.

Adult size is a critical economic trait for all aquaculture species^14^, as larger fish are more valuable to consumers. Thus, the development of broodstock that can grow rapidly is of great interest. However, genomic resources for *L. polyactis* are not well developed*,* limiting our understanding of genes that contribute to growth in this species. Growth is a quantitative trait controlled by multiple genes, or quantitative trait loci (QTLs). Compared to traditional selective breeding programs that focus in selecting individuals with desirable traits, molecular based breeding programs accelerate the breeding process by using genetic markers that can be quantified faster than adult traits^15^. Dense genetic maps are necessary for molecular breeding.

The development of next-generation sequencing (NGS) technologies have facilitated the sequencing and assembly of many fish genomes, such as *Cynoglossus semilaevis*^16^, *Cyprinus carpio*^17^, *Larimichthys crocea*^18^, *Miichthys miiuy*^19^, *Paralichthys olivaceus*^20^, *Lateolabrax maculatus*^21^, *Epinephelus lanceolatus*^22^, and *Carassius auratus*^23^. Recently, the first reference genome of *L. polyactis* was sequenced and assembled (Xie et al., unpublished), and genome-scale SNP markers can now be identified through resequencing of the genome, which allows for the construction of high-density linkage maps. Genetic linkage maps are essential resources that can improve the quality of the genome and chromosomal assembly^24^, and are essential for mapping QTLs. QTL mapping identifies genes that are related to phenotype variations within populations, and genes identified through QTL studies could be used to enhance breeding programs.

The objectives of this study were: (1) large-scale identification of SNPs and construction of a high-density SNP-based linkage map using genotyping by resequencing individuals of *L. polyactis*; (2) QTL mapping to detect SNPs associated with growth traits using the high-density map; and (3) identification of candidate genes that regulate growth related-traits of *L. polyactis*. This study establishes large-scale SNP markers and high-density linkage maps for *L. polyactis*, which can facilitate genome assembly, comparative genomics, and QTL mapping for economic traits in *L. polyactis.*

## Results

### Values of the phenotypic traits

We used 110 *L. polyactis* progeny to generate the linkage map. The average values for total length, body length, and body weight were 16.62 ± 1.10 cm, 13.76 ± 0.87 cm, and 48.89 ± 10.33 g, respectively. These growth traits were strongly correlated with each other (r = 0.902–0.949, *P* < 0.01 for all) (Table 1). Body length and body weight had the highest correlation (r = 0.949). Of the progeny, 35 and 66 individuals were identified as males and females, respectively, with a sex ratio of 1:1.89. We were unable to identify the sex of the remaining nine individuals of the 110 progeny.

**Table 1:** Pearson correlation coefficients (r) for all pairwise combinations of the three growth-related traits (*P* < 0.01 for all).

	Total length	Body length	Body weight
Total length	1		
Body length	0.946	1	
Body weight	0.902	0.949	1

### Construction of sequencing library and sequencing

A total of 112 sequencing libraries from two parents and 110 progenies were constructed and sequenced to generate 849.37 Gb of sequencing data, which resulted in 847.47 Gb of high-quality sequencing data with an average Q20 ratio of 97.09% and a GC content of 42.28%. The parents were sequenced at a higher level to enhance the chances of detecting more SNP markers. Finally, clean data covering 14.78 Gb with a GC% of 42.10 and 13.56 Gb with a GC% of 42.33 were obtained for the male and female parents, respectively. For each individual, the clean data ranged from 6.32 to 9.89 Gb, with an average of 7.45 Gb (Supplementary Table S1). From the paired end clean reads, 97,046,018 reads were obtained from the female parent and 105,785,790 reads were obtained from the male parent. Only reads that aligned to unique positions on the reference genome were retained for the subsequent SNP calling and genotyping (Supplementary Table S2).

### SNP discovery and genotyping

SNP calling of the two parents and F1 individuals was performed using GATK. A total of 777,570,223 markers were detected, of which 14,317,836 were retained once SNPs with more than 20% missing data in both genotype and individual were removed. After initial genotyping, the CP model was used in JoinMap5.0 to select for segregation types lm × ll (48,698 markers), nn × np (47,340 markers), and hk × hk (28,809 markers), which resulted in a total of 124,847 SNPs that were polymorphic in at least one parent and 92% of progeny (Table 2).

**Table 2:** Number of genotyping SNPs in each segregation types in F_1_ individuals.

Marker type	Mother genotype	Father genotype	Marker number	Percentage
hkxhk	hk	hk	28,809	23.07%
nnxnp	nn	np	47,340	37.92%
lmxll	lm	ll	48,698	39.01%
Total			124,847	100.00%

### Construction of the genetic map

**Figure 1:**
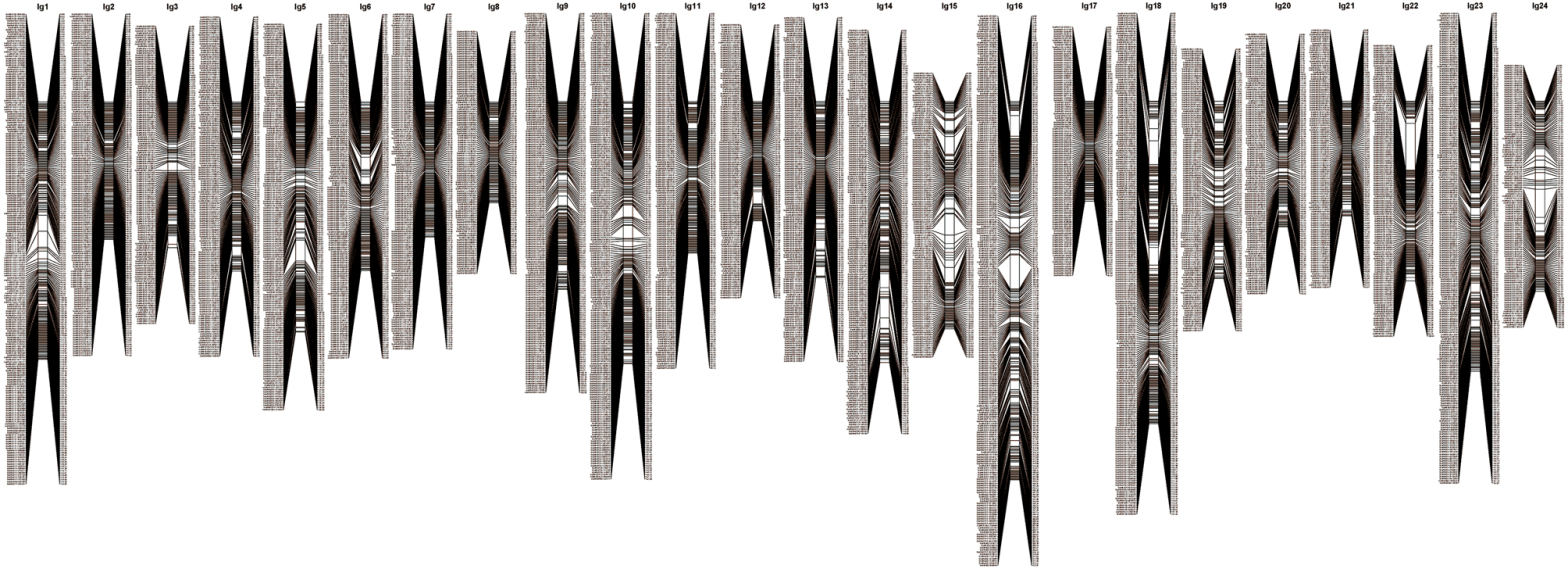
The consensus linkage map of L. polyactis. The consensus map which contained 3,802 effective SNPs in 24 linkage groups was constructed through combing the male and female linkage maps.

Once SNPs with significant segregation distortion were removed, 94,169 SNPs were retained to construct the genetic map. Linkage group (LG) assignments were made using the separate chromosomes module with a logarithm of odds (LOD) score limit of 12. On the female map, 49,235 SNPs were categorized into 24 linkage groups (LGs). Subsequently, the redundant SNPs that mapped to the same loci were removed, and 1,299 effective SNPs were selected with a genetic length of 1942.42 cm and an average distance of 1.51 cm (Supplementary Fig. S1). On the male map, 49,074 SNPs were categorized into 24 LGs, and 1,405 effective SNPs were selected with a genetic length of 1773.64 cm and an average distance of 1.27 cm (Supplementary Fig. S2). The two parent maps were merged and redundant SNPs were removed, resulting in the integrated map that spanned 2,567.39 cm with 3,802 effective SNPs in 24 LGs (Fig. 1; Supplementary Fig. S3). Among the 24 LGs in the integrated map, LG16 was the largest group with a genetic distance of 212.87 cm and 249 effective SNPs, and LG17 was the shortest with 113 markers spanning 56.22 cm. The average effective SNP interval ranged from 0.50 to 0.99 cm, with an average distance of 0.66 cm (Table 3; Supplementary Table S3).

**Table 3:** Characteristics of genetic maps and anchoring scaffolds of *L. polyactis.*

Linkage	Maternal map		Paternal map		Integrated map		
	No. of SNPs/effective SNPs	Distance (cm)	No. of SNPs/effective SNPs	Distance (cm)	No. of SNPs/effective SNPs	Distance (cm)	Effective average distance (cm)
LG1	2,519/58	61.52	2,861/70	131.9	5,317/213	144.85	0.68
LG2	2,528/53	70.76	2,927/67	74.43	4,937/155	77.25	0.50
LG3	2,452/59	77.56	2,638/51	56.99	4,783/135	81.85	0.61
LG4	2,767/53	84.69	2,578/60	69.81	4,925/154	95.10	0.62
LG5	2,582/66	99.05	2,267/62	62.84	4,543/175	129.36	0.74
LG6	2,322/57	69.93	2,268/65	68.88	4,298/156	94.80	0.61
LG7	2,329/69	83.70	2,332/77	88.25	4,299/152	75.87	0.50
LG8	2,322/39	54.37	2,111/49	51.43	4,147/110	56.69	0.52
LG9	2,495/67	96.83	1,958/62	82.04	4,182/172	105.68	0.61
LG10	2,146/61	93.79	2,232/53	66.25	4,076/211	147.6	0.70
LG11	2,104/51	91.46	2,161/62	67.02	3,980/161	85.14	0.53
LG12	1852/47	71.27	2,045/52	68.09	3,660/124	67.14	0.54
LG13	2000/51	70.15	1,932/68	82.12	3,630/156	98.99	0.63
LG14	1898/62	76.71	1,836/54	68.37	3,411/183	146.88	0.80
LG15	1959/55	159.06	1,679/40	71.03	3,430/129	127.83	0.99
LG16	1602/70	110.19	1,996/67	98.50	3,300/249	212.87	0.85
LG17	2,347/51	56.14	1,931/63	56.88	3,438/113	56.22	0.50
LG18	1672/54	103.82	1,933/56	71.66	3,410/227	181.55	0.80
LG19	1835/46	86.89	1,729/59	68.25	3,331/128	99.70	0.78
LG20	1517/41	75.35	1,630/68	113.88	2,947/118	70.59	0.60
LG21	1,610/40	57.33	1,656/45	54.16	3,044/117	64.57	0.55
LG22	1652/53	75.84	1,526/52	57.02	2,798/132	87.87	0.67
LG23	1,419/59	62.50	1,520/67	88.51	2,689/213	152.07	0.71
LG24	1,306/37	53.51	1,328/36	55.33	2,483/119	106.92	0.90
Total	49,235/1,299	1942.42	49,074/1,405	1773.64	91,058/3,802	2,567.39	-
Average	2051.46/54.13	80.93	2,044.75/58.54	73.90	3,794.08/158.42	106.97	0.66

### QTL mapping of growth traits

The integrated linkage map with 3,802 effective SNPs in 24 LGs of *L. polyactis* was used to identify QTLs that associate with growth traits. The interval mapping model in the MapQTL package was used to analyze the total length, body length, body weight of 110 progeny. The estimated significant thresholds from permutation tests were 4.5 for total length, 4.8 for body length, and 3.9 for body weight.

**Figure 2:**
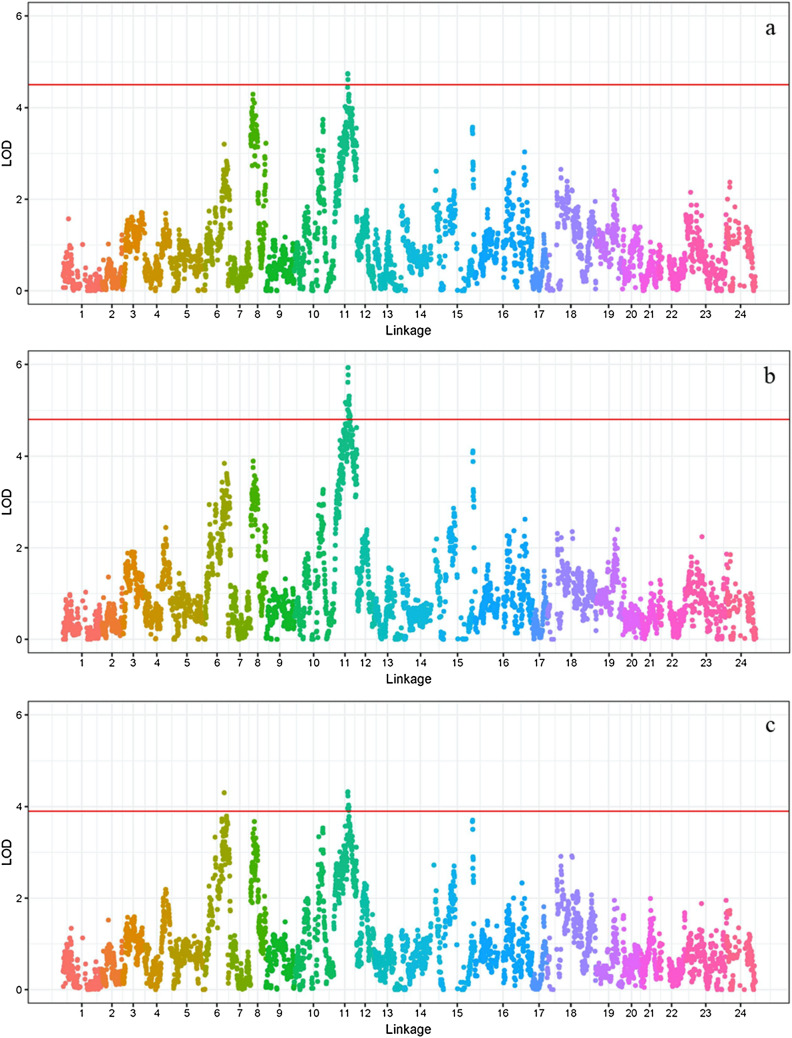
Total length (**a**), body length (**b**) and body weight (**c**) QTL mapping and association analysis in *L. polyactis* among all linkage groups. The x- and y-axes correspond respectively to relative position on the linkage groups and the LOD value. The red horizontal line represents a linkage group-wise logarithm of odds (LOD) significance threshold of 4.8 for total length, 4.5 for body length, and 3.9 for body weight.

The numbers of SNPs that were detected for each trait are shown in Table 4. We found three significant SNPs for total length with LOD scores of 4.74, 4.73 and 4.61, and these SNPs were located at 51.77, 52.23 and 52.69 of LG11. The proportion of phenotypic variation explained by these SNPs was 18.5%, 18.5% and 18.1%, respectively. Thus, this is a candidate genomic region for controlling total length of *L. polyactis* (Fig. 2a; Supplementary Fig. S4; Supplementary Table S4). Similar to total length, body length had 19 significant SNPs in LG11, and their LOD values were larger than 4.5. The proportion of phenotypic variation explained by these SNPs ranged from 18.30 to 22.6%. Thus, this region is a candidate genomic region for determination of body length of *L. polyactis* (Fig. 2b; Supplementary Fig. S5; Supplementary Table S4). Nine SNPs were associated with body weight, and the proportion of phenotypic variation explained by these SNP ranged from 15.20 to 17.10%. One SNP of the nine was detected in LG6, and the rest were located in LG11, thus these two regions represent candidate regions for determining body weight in *L. polyactis* (Fig. 2c; Supplementary Fig. S6; Supplementary Table S4). Interestingly, three SNPs in LG11 were shared among total length, body length and body weight. In addition, eight SNPs were shared for both body length and body weight.

**Table 4:** Detected QTLs for growth-related traits of *L. polyactis.*

Traits	Linkage	Position	Number of significant SNPs	LOD threshold value	LOD value of detected SNPs	Exp%^a^
Total length	LG11	51.77–52.69	3	4.5	4.61–4.74	18.10–18.50
Body length	LG11	42.61	1	4.8	5.17	19.50
		46.75	1		5.13	19.40
		50.86–52.69	5		5.01–5.93	19.40–22.60
		54.06–58.17	10		4.81–5.31	18.3–20.10
		59.55–60.00	2		4.87–4.89	18.50–18.60
Body weight	LG6	71.93	1	3.9	4.30	16.9
	LG11	51.32–52.69	4		3.96–4.32	16.40–17.10
	LG11	54.98–56.35	4		3.91–4.03	15.20–15.70

^a^ percentage of explained phenotypic variation.

### Potential candidate genes

To further identify potential causative genes underlying growth traits, we screened the reference genome and identified protein-coding genes by mapping the corresponding tags of SNPs in QTL regions to the scaffold assembly followed by retrieval of the corresponding gene from the gene annotation file. Thirteen genes were thus identified, including kinesin-like protein KIF26B (*kif26b*), E3 ubiquitin-protein ligase AMFR-like (*amfr*), Nucleolar RNA helicase 2 (*ddx21*), elongation factor for RNA polymerase II (*ell*), polypeptide N-acetylgalactosaminyltransferase 2 (*galnt2*), globoside alpha-1,3-N-acetylgalactosaminyltransferase 1 (*gbgt1*), glucosamine 6-phosphate N-acetyltransferase (*gna1*), Homeodomain-interacting protein kinase 3 (*hipk*), solute carrier family 6 member 2 (*slc6a2*), B(0, +)-type amino acid transporter 1 (*bat1*), serine/threonine/tyrosine interacting protein (*styx*), ArfGAP with GTPase domain, ankyrin repeat and PH domain 2 (*agap2*) (Supplementary Table S5). Of these, the most significant SNP located in the QTLs on LG11 that was associated with total length, body length and body weight revealed *kif26b* as a potential candidate gene that may regulate growth in *L. polyactis.* The candidate genes of *amfr*, *ddx21*, *gbgt1*, *gna1*, *bat1*, *styx* detected on LG11 were also shared for body length and body weight. These candidate genes may contribute to the genetic control of growth traits. The exact function of these genes in *L. polyactis* needs further study.

## Discussion

### High density genetic linkage map

Since the publication of the first genetic linkage map of tilapia in 1998^25^, various linkage maps have been developed for aquaculture species using microsatellites, as well as SNPs. Compared to microsatellites with long flanking DNA sequences, SNP markers that are most abundant in an organism have been more suitable for genetic studies^26^, especially in high-density genetic linkage mapping^27^, QTL mapping^28^, population genetic analysis^29^, and genome-wide association studies (GWAS)^30^. In the past, developing SNP markers and studying their patterns in large populations were technologically challenging and expensive. Today, sequencing an individual’s genotype is simple, quick, and reproducible^31^, and a number of high-density linkage maps have been constructed for aquaculture species, such as *P. olivaceus*^32^, *Lates calcarifer* ^33,34^, *C. carpio*^35^, *C. auratus*^36,37^, *Megalobrama amblycephala*^38^, and *Colossoma macropomum*^39^. Here, we constructed the first genetic map of *L. polyactis* using 94,169 SNPs, and the integrated map consists of 24 LGs, which corresponded to 3,802 effective loci. The total length of the integrated map was 3,794.08 cm, with an average inter-locus distance of 0.66 cm. The value of the average locus distance was larger than *P. olivaceus* (0.47)^32^, *L. crocea* (0.54)^40^, *C. auratus* (0.44)^36^, *Seriola aureovittata* (0.30)^41^, but smaller than *Dicentrarchus labrax* (0.72)^42^, *Hypophthalmichthys nobilis* (0.75)^43^, *C. carpio* (0.75)^35^, *Scophthalmus maximus* (0.72)^44^, and *Silurus meridionalis* (0.89)^45^. Compared with other fish genetic maps, our genetic map is relatively high in quality. The high quality linkage map that we generated has enabled us to determine the number and position of the QTLs for growth traits, and will allow for fine-mapping of QTLs in molecular breeding of *L. polyactis*. We also constructed the paternal and maternal linkage maps of the *L. polyactis,* and these data are valuable resources for genome selection (GS) and GWAS in *L. polyactis*. Due to the limited the number of individuals that were sequenced, and the use of the F_1_ generation as the mapping group, the number of non-redundant markers that mapped onto the map was relatively small. Therefore, in the subsequent research, the F_2_ generation should be used as the mapping group, and more than 200 individuals should be genotyped.

### Candidate growth-related genes

To improve aquaculture of *L. polyactis*, it is important to identify markers linked to economically important traits to breed superior parental stock. QTL analyses enable us to find the markers associated with the genetic variation for growth-related traits and the candidate genes involving physiological processes for the traits, which can be applied to breeding programs. We found strong correlations (r ≥ 0.902) among the three growth-related traits that were measured, and this finding was similar to previous work on other aquaculture species^46–48^. QTL analyses of growth-related traits have been conducted in some aquaculture species, such as *Oncorhynchus mykiss*^49^, *Salmo salar*^50^, *P. olivaceus*^51^, *C. semilaevis*^52^, tilapia^53^, and *L. calcarifer*^33^. As growth traits are quantitative traits controlled by multiple genes, growth-related QTLs are mapped to several LGs in most cases. For example, O’Malley et al.^54^ identified QTLs for body weight in rainbow trout on 10 different LGs, Gutierrez et al.^50^ and Reid et al.^55^ identified QTLs for body weight in two LGs and six LGs of Atlantic salmon, respectively. In the present study, multiple QTLs were found on two LGs for growth-related traits, and three SNPs on LG11 were shared for total length, body length and body weight, suggesting that this QTL affects all three growth-related traits in *L. polyactis*. Eight SNPs that were associated with body weight and body length had the same distribution pattern on LG11. One SNP detected for body weight was located on LG6, and this SNP was unique to body weight. In all, QTLs related to different growth traits were generally located on the same LGs, though a few were found on different LGs.

QTL mapping enables us not only to detect genetic markers associated with important traits, but also to identify candidate genes that regulate traits of interest^56^. For example, Wang et al.^57^ identified *cathepsin D*, *KCTD15*, and *csmd2* as genes that affect body weight, body length, and total length of the Asian seabass using QTL mapping^58^. Wringe et al.^49^ found several major candidate growth genes (e.g., *GH2* and *Pax7*) in *Oncorhynchus mykiss*. Tao and Boulding^58^ found *GH* was significantly associated with growth rate of *Salvelinus alpinus*. Sun et al.^59^ identified two SNPs in *MSTN* that were significantly associated with body weight and Fulton’s factor in the common carp. Liu et al.^60^ identified a SNP in the UTR of *MSTN* 3′ that was strongly associated with total length, body length, and body weight of *Aristichthys nobilis*. In the present study, candidate genes identified from the growth-related QTLs may play critical roles in the genetic regulation of development, cell-proliferation, energy metabolism, and immunity. For example, *kif26b* was identified from the significant QTL intervals on LG11 as a candidate gene that was consistently associated with all three growth traits. As a kinesin family gene, *kif26b* plays an important role in the compact adhesion between mesenchymal cells^61^. It was also reported that overexpressing *kif26b* promoted cell proliferation and migration, while silencing of *kif26b* had the opposite effect^62,63^. Therefore, we hypothesized that *kif26b* may regulate growth traits through promoting cell proliferation. Another candidate gene, *bat1*, associated with body length and body weight, belongs to the family of amino acid transporters associated with type II membrane glycoproteins^64^, and plays an important role in the protein digestion and absorption pathway. The candidate gene, *gna1*, also associated with body length and body weight, is a key enzyme in the pathway for biosynthesis of UDP-N-acetylglucosamine, an important donor substrate for N-linked glycosylation^65^, which is important in amino sugar and nucleotide sugar metabolism. The gene *gbgt1*, which encodes the globoside alpha-1,3-N-acetylgalactosaminyltransferase 1, regulates glycosphingolipid biosynthesis^66^, which is important in glycan biosynthesis and metabolism. Finally, *amfr* is a ubiquitin protein ligase implicated in protein processing in the endoplasmic reticulum^67^. According to previous reports, we speculated that these genes may be involved in the growth regulation of *L. polyactis*, which requires further careful study to be verified. In addition, there are likely to be other undetected genes that regulate growth-related traits that need to be studied further using a genetic map with a higher density of SNPs.

## Material and methods

### Mapping family and DNA extraction

A F_1_ full-sib family of *L. polyactis* was generated for genetic mapping at the breeding station of Marine Fishery Institute of Zhejiang Province (Xishan Island, Zhoushan, China) in April 2017. Larva was cultured as described in Liu et al.^2^. The female and male parents were from Xiangshan Harbour wild stocks. Muscle samples of the parents were collected and stored in 100% ethanol, and kept in a − 20 °C freezer. Seven months after hatching, 110 full-sibling offspring were randomly sampled for linkage analysis, and total length (cm), body length (cm), and body weight (g) were measured. The sex of each individual was identified based on anatomical observation of gonads. Muscle tissue from each individual was collected and preserved in 100% ethanol for DNA extraction.

Genomic DNA was extracted using the TIANGEN Marine animal DNA extraction kit (TIANGEN, Beijing, China). The concentration of the extracted genomic DNA was quantified using a NanoDrop 2000 (Thermo, USA) and the DNA integrity of each individual sample was evaluated by 1% agarose gel electrophoresis. DNA samples were stored at − 20 °C for further experiments.

### Sequencing library preparation and next generation sequencing

SNP identification and genotyping were performed by resequencing of the genome of *L. polyactis* individuals. Sequencing libraries were constructed following Nunes et al.^39^ with slight modifications. Genomic DNA of each individual was fragmented into about 350 bp fragments by a Covaris crusher. Sequencing libraries were generated using a TruSeq Library Construction Kit following the manufacturer's instructions. Digested DNA fragments were examined by electrophoresis and were then ligated with barcode adaptors. Ligation products were pooled to select for 350–600 bp fragments using Pippin Prep (Sage Science, USA) after clean up with a QIAquick PCR Purification Kit (Qiagen, Germany). Libraries were enriched by PCR using Phusion® High-Fidelity DNA Polymerase (New England Biolabs, USA). Finally, libraries were cleaned using the QIAquick PCR Purification Kit (Qiagen, Germany) and quantified using the KAPA Library Quantification Kit (Kapa Biosystems, USA) for paired-end sequencing on an Illumina HiSeq™ PE150 platform (Illumina, USA), which produced single-end raw reads of 150 bp.

### Sequence data analysis and genotyping of SNPs

Barcodes were used to sort the raw reads from each individual. To ensure that reads were reliable, raw data (raw reads) in FASTQ format were processed through a series of quality control (QC) procedures with in-house C scripts. QC standards were as follows: (1) reads with ≥ 10% unidentified nucleotides (Ns) were removed, (2) reads containing the HaeIII or EcoRI sequences were removed, and (3) reads with > 50% of bases having a Phred quality < 5 were removed. The clean reads from each individual were aligned against the reference genome (Xie et al., unpublished) using the Burrows-Wheeler Aligner (BWA) software (settings: mem -t 4 -k 32 -M -R). If multiple read pairs had identical external coordinates, the pair with the highest mapping quality was retained. Alignment files were converted to bam files using SAMtools^68^ (settings: –bS –t). When multiple read pairs had identical external coordinates, the pair with the highest mapping quality was retained. SNP calling was performed for parents and progeny using the Broad Institute’s open-source GATK software (-type UnifiedGenotyper). Unreliable SNPs were eliminated by a filtering process. A Perl script was used to filter the SNPs that had more than two genotypes. Any SNP with more than 20% missing data in both genotype and individual were removed from further analysis.

### Linkage map construction

Linkage analysis was conducted by taking a pseudo-test cross strategy. SNPs were divided into three categories according to their segregation patterns: AB × AA or AB × BB (1:1 segregation only in female parent), AA × AB or BB × AB (1:1 segregation only in male parent), and AB × AB (1:2:1 segregation in both parents). Markers showing significant segregation distortion (*P* < 0.001) were removed. The remaining SNPs were used to construct the genetic map with the package Lep-MAP (https://sourceforge.net/projects/lep-map3/)^69^. Genetic maps were constructed following methods described in Shao et al.^32^. The genotype data were filtered manually to remove obvious Mendelian errors from the offspring. The group LOD value ranged from 4 to 20, depending on the linkage group. Maps were generated by regression mapping using Lep-MAP and the Kosambi mapping function in Lep-MAP was used to convert the recombination frequencies into map distances in centiMorgans (cm). The order of markers was obtained using the order markers module of Lep-MAP. An integrated map was constructed using male and female maps that were originally generated using only one input file. To speed up the computation, constant rates for genotype errors and recombination were used. Finally, marker positions were established using the order markers module.

### QTL mapping for growth traits

Pearson’s correlations of the three growth-related traits were performed for all progeny. The QTLs of growth traits were identified using MapQTL 5.0^70^ using Interval Mapping (IM). Automatic cofactor selection (backward elimination, *P* < 0.05) was used to select significantly associated markers as cofactors. The LOD significance threshold levels were determined by Permutation Tests with 1,000 permutations at significance level of *P* < 0.05. QTLs with LOD scores exceeding the genome-wide LOD threshold at *P* < 0.05 were considered to be significant. The location of each QTL was determined based on its LOD peak location and the surrounding region. MapQTL 5.0 was used to identify QTL reference values, including phenotypic variation and positive or negative additive effect for growth traits. The candidate genes in the QTL intervals were identified based on the genome annotation information, and the genes that the SNPs of interest located in were regarded as candidate genes of particular interest.

### Ethics statement

This study was approved by the Animal Care and Use committee of Centre for Applied Aquatic Genomics at Zhejiang Academy of Agricultural Sciences. The methods were carried out in accordance with the approved guidelines.

## Supplementary information

Supplementary Figures.

Supplementary Table S1.

Supplementary Table S2.

Supplementary Table S3.

Supplementary Table S4.

Supplementary Table S5.
