# Supplementary material for: A first genetic linage map construction and QTL mapping for growth traits in Larimichthys polyactis
Source: Sci Rep. 2020 Jul 15;10:11621. doi: 10.1038/s41598-020-68592-0 (PMC7363912; doi:10.1038/s41598-020-68592-0)
Supplement: Supplementary file 1 — Supplementary Figures. [file 41598_2020_68592_MOESM1_ESM.docx]

**A first genetic linage map construction and QTL mapping for growth traits in *Larimichthys polyactis***

Feng Liu^1^*, Wei Zhan^1^, Qingping Xie^1^, Honglin Chen^1^, Bao Lou^1^*, Wantu Xu^2^

^1^ Institute of Hydrobiology, Zhejiang Academy of Agricultural Sciences, Hangzhou 310021, China

^2^ Xiangshan Gangwan Aquatic Seeds Co., Ltd., Ningbo 315700, China


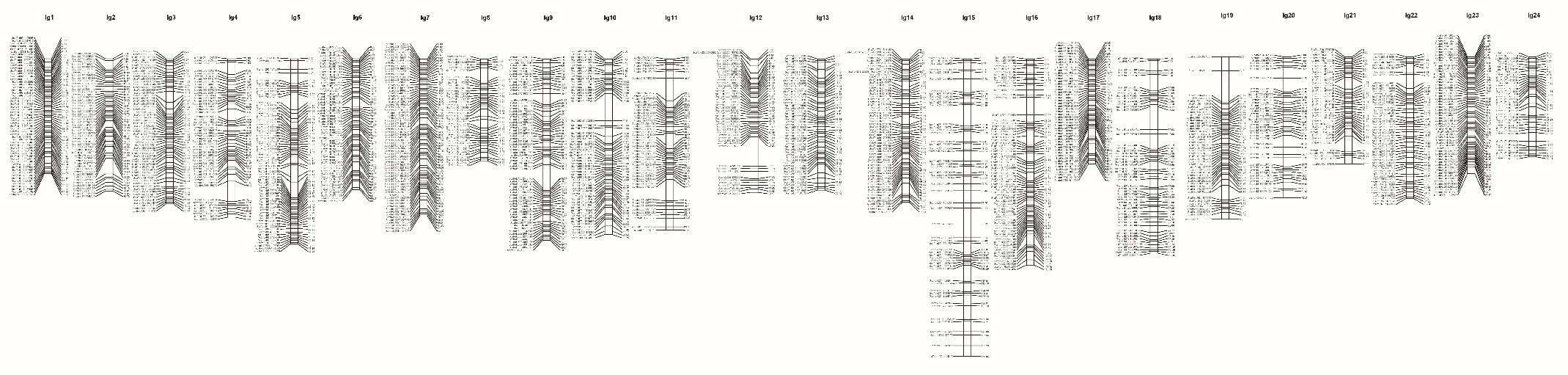


Figure S1 The female-specific linkage map of *L. polyactis*.


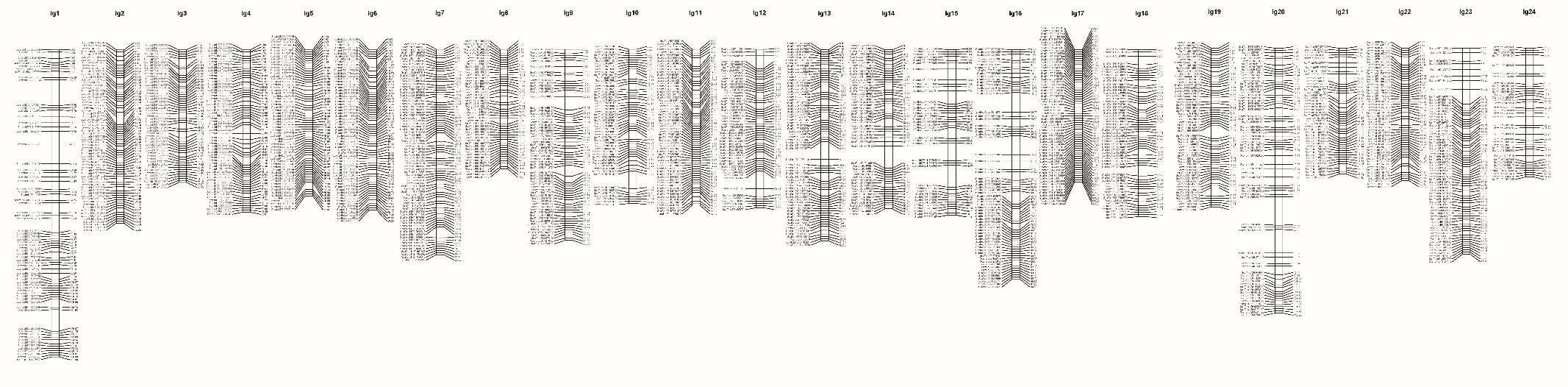


Figure S2 The male-specific linkage map of *L. polyactis*.


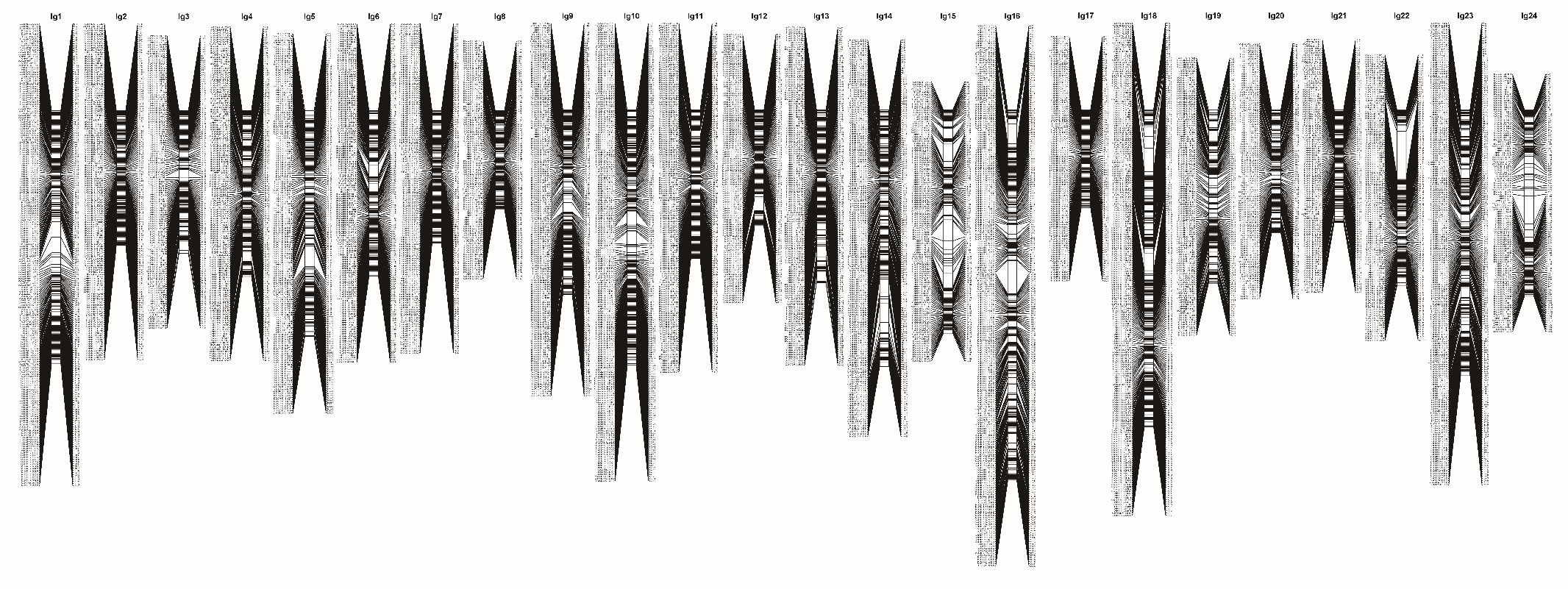


Figure S3 The consensus genetic linkage map of *L. polyactis*. The consensus map which contained 3802 effective SNPs in 24 linkage groups was constructed through combing the male and female linkage maps.


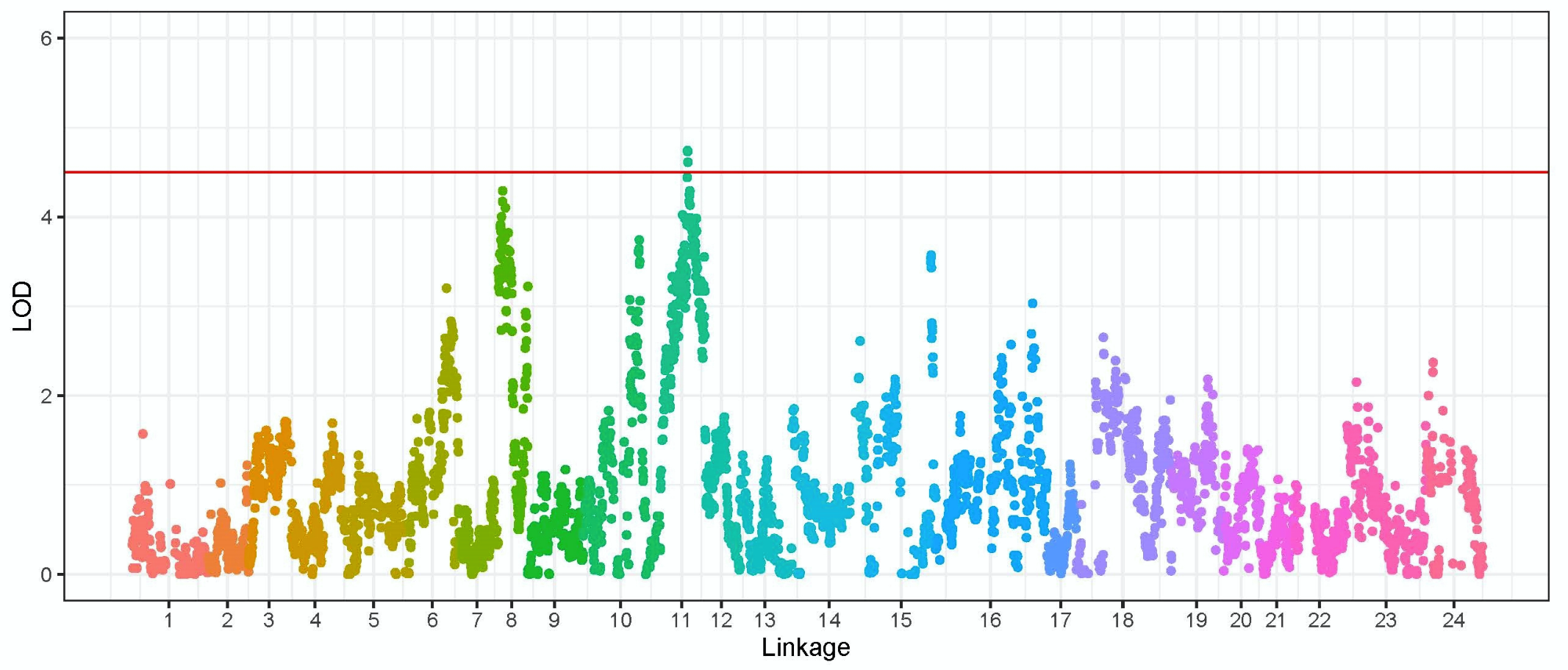


Figure S4 Total length QTL mapping and association analysis in *L. polyactis* among all linkage groups. The x- and y-axes correspond respectively to relative position on the linkage groups and the LOD value. The red horizontal line represents a linkage group-wise logarithm of odds (LOD) significance threshold of 4.8.


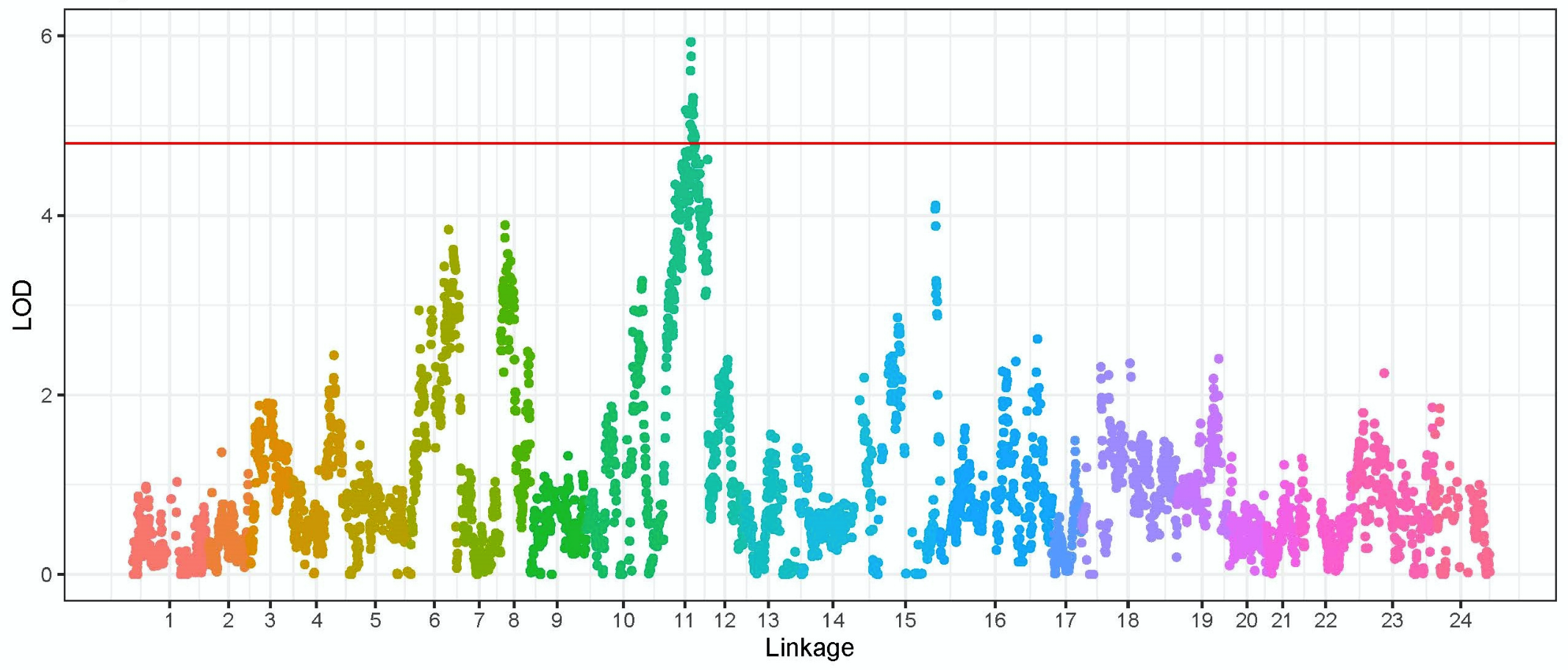


Figure S5 Body length QTL mapping and association analysis in *L. polyactis* among all linkage groups. The x- and y-axes correspond respectively to relative position on the linkage groups and the LOD value. The red horizontal line represents a linkage group-wise logarithm of odds (LOD) significance threshold of 4.5.


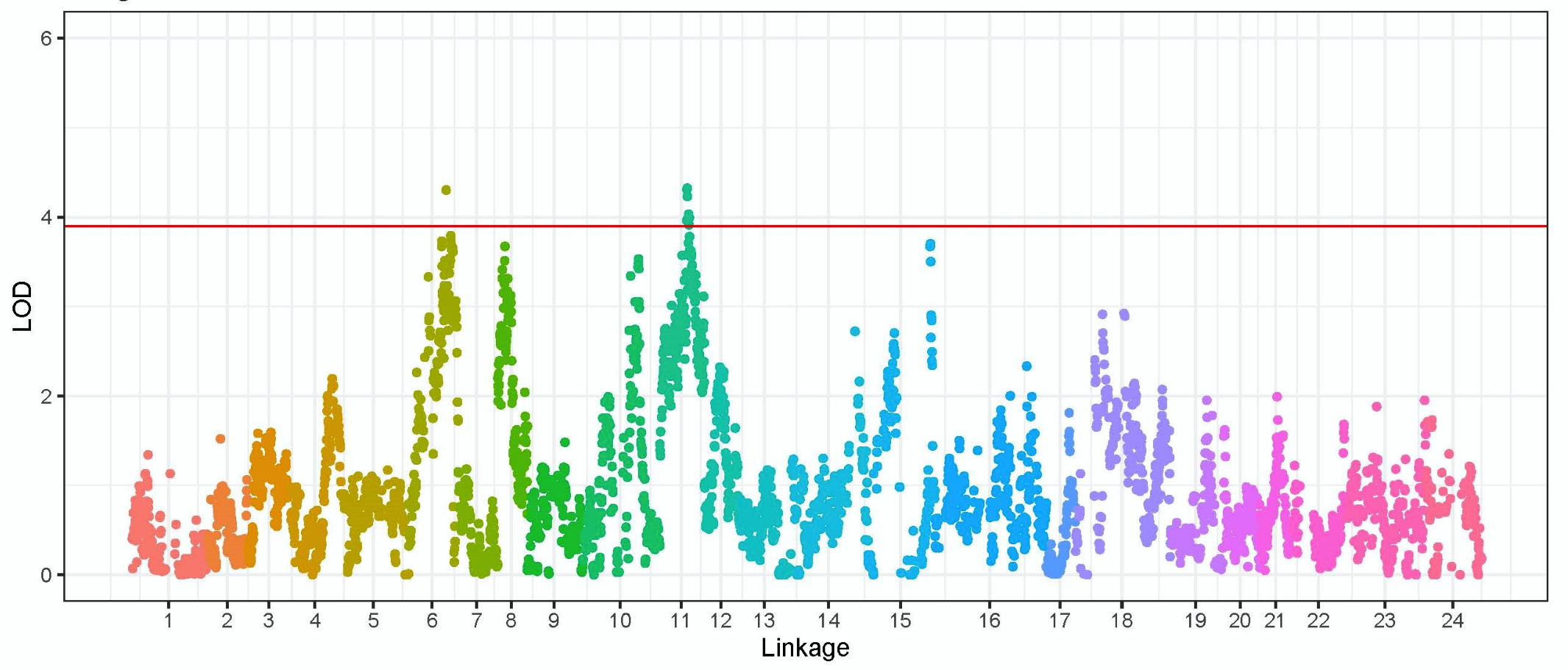


Figure S6 Body weight QTL mapping and association analysis in *L. polyactis* among all linkage groups. The x- and y-axes correspond respectively to relative position on the linkage groups and the LOD value. The red horizontal line represents a linkage group-wise logarithm of odds (LOD) significance threshold of 3.9.
